# Elizabeth Schuyler Hamilton in psychobiography: Sense of coherence and faith across her lifetime

**DOI:** 10.3389/fpsyg.2022.948167

**Published:** 2022-08-22

**Authors:** Claude-Hélène Mayer

**Affiliations:** Department of Industrial Psychology and People Management, University of Johannesburg, Johannesburg, South Africa

**Keywords:** coping, sense of coherence, faith development theory (FDT), positive psychology (PP), women in psychobiography, USA women leaders

## Abstract

This article focuses on the coping skills of one selected, extraordinary woman, Elizabeth Schuyler Hamilton (1757–1854) during the founding of the United States of America. This work contributes to theory on two different levels. First, it contributes to psychobiographical research on women in diverse spheres of society, thereby strengthening the aspect of gender and coping strategies in terms of psychobiographical perspectives. Second, it contributes to theory-building in psychobiographical research anchored in positive psychology, promoting the idea that a multiplicity (crystallisation) of theories should be used to explore and analyse the lifespan of extraordinary individuals. The psychobiography responds to the question how Elizabeth Schuyler Hamilton coped with life’s challenges and tragedies through the lenses of sense of coherence and faith development theory. The article uses a psychobiographical case study design within the research paradigm of modern hermeneutics. First- and third-person data on the subject were collected and evaluated through thematic analysis, including articles, documentation, letters, film material, and political scripts. Customary ethical standards for psychobiographical research were followed, thereby ensuring an ethical, respectful, empathetic and accountable research approach. The article presents findings on the coping skills, sense of coherence, and faith development in the life of Elizabeth Schuyler Hamilton which strongly influenced her resilience and support for others during her long and extraordinary life. Conclusions are drawn with regard to the way women cope in different sociocultural, sociopolitical and socio-economic spheres using historical and contemporary retrospectives. Recommendations are provided for future psychobiographical research on women in diverse contexts and in psychobiographical, gendered practice.

*Look around*, *look around*, *how happy we are*

*to be alive right now.*

*Elizabeth Schuyler Hamilton*

## Introduction

Previous research, both historical and contemporary, has shown that in comparison to men, women leaders are seldom recognised for their strong contributions to leadership, organisational success, social change and welfare, and societal flourishing (Milazzo and Goldstein, 2019; Mayer, 2022; Women U. N., 2022). Often, they have remained unacknowledged and “invisible” in supporting their husbands’ careers and affecting different spheres of society (Christensen, 2019). However, recent research shows that these women were often supporters of their husbands, typists of their written accounts, defenders of their work, assistants, muses and managers who contributed to the work of their husbands in creative and intellectual ways, although often hidden and suppressed (Christensen, 2019). Simonton (1996) has pointed out that presidents’ wives (First Ladies) often gain distinction through their relationships with their husbands while staying in traditional gender roles. In history and until recently, there has been limited research on the wives of presidents and other famous men (Whitton, 1948; Barzman, 1970; Caroli, 1987; Boller, 1988). However, being in the role of the daughter or wife of a famous man comes with challenges (Wade, 2021). Oakley (2021) emphasises that women are often reduced to gendered roles and stereotypes and live accordingly. In her research, Oakley (2021) analyses the lives of four wives of famous British men in the middle and upper classes during the 21st century and highlights how marriage determines the economic position, psychology, physical and mental health of men and women in different ways. Through marriage, wifehood becomes a political and institutionalised status in which women are often “forgotten” – a term that includes aspects of being ignored, devalued, marginalised and distorted (Oakley, 2021, p. 3).

Elizabeth Schuyler Hamilton (ESH; 1757–1854), nicknamed Eliza or Betsey, was an extraordinary woman who first became famous through marriage to one of the founding fathers of the United States of America (US), Alexander Hamilton (Miller, 1959; Knott, 2002; Chernow, 2004; Miranda, 2015). She married Hamilton when she was 23, and was a great supporter of his political work during and beyond his lifetime. She also supported his political career, negotiated for him with his publishers, raised their eight children, and paid off his debts after his death. Furthermore, she helped to raise funds for the Washington monument, and co-founded the first private orphanage in New York City (Kenyes, 1931; New Netherland Institute, 2020). She was often referred to in her role as Hamilton’s wife (Randall, 2003; Chernow, 2004), although several brief biographies exist on her life as a philanthropist and social activist (Presnell, 2004; History of American Women, 2022). Nevertheless, Presnell (2004) observes that most of the information on her needs to be extracted from biographies written about her husband (McDonald, 1979; Hendrickson, 1981; Emery, 1982) and these often only mention her briefly (Miller, 1959; Knott, 2002).

The present psychobiographical account focuses on the life of ESH, who managed to cope with many challenges and tragedies in her life – such as the early death of her first-born son and her husband – and to remain “incredibly strong of spirit” (Esme, 2022).

In this article, the theoretical foundation used to analyse the coping skills of ESH during her lifetime, is based on two theoretical approaches which are part of the positive psychology theoretical paradigm: the salutogenetic theory, and in particular the sense of coherence (SOC) of Antonovsky (1979), and Fowler’s faith development theory (FDT) of 1981 and 1984. Both of the theories have been previously applied to psychobiography. Salutogenesis and the exploration of SOC were used in the psychobiography of Steve Jobs (Moore, 2014) and of Viktor von Weizsäcker (Mayer and Bahrs, 2021). The FDT has been used in psychobiographies such as those of Mother Theresa (Stroud, 2004), Beyers Naude (Burnell, 2013; Fouché et al., 2016), Anne Hutchinson, Ludwig Wittgenstein, Dietrich Bonhoeffer, Blaise Pascal and Malcom X (Fowler and Lovin, 1979), John Wesley (Fowler, 2001), Paulo Coelho (Mayer, 2017), Barach Spinoza (Mayer and Fouché, 2021) and Angela Merkel (Mayer, 2021a).

This study contributes to the psychobiographical literature on women by making the life and coping skills of one selected woman more “visible” through salutogenesis and SOC, as well as the development of faith in coping with all of her challenges. Coping is thereby viewed as an interactional process between the individual person researched and her environment (Lazarus and Folkman, 1984). It has been defined as the effort to exert by individuals to deal with demands from the environment to reduce stress and contribute to individual health and well-being (Braun-Lewensohn and Mayer, 2020). Coping includes also the cognitive process to deal with stress and challenges (Lazarus and Folkman, 1984). Salutogenesis and faith can be seen as resources to cope with life’s challenges, as shown in previous psychobiographical and mixed-method studies (Mayer, 2017; Mayer and Fouché, 2021; Mayer et al., 2021a).

This psychobiography further contributes to positive psychology accounts in psychobiography by using two theories which are anchored in a positive psychology framework (Mayer, 2017, 2021a). The main research question which this psychobiography responds to is: *“How did Elizabeth Schuyler Hamilton cope with life’s challenges and tragedies through the lenses of sense of coherence and faith development theory?”* The question “how” ESH coped is here of utmost importance to explore the coping mechanisms and skills, as shown in previous psychobiographical research (Mayer et al., 2021b).

## Salutogenesis and sense of coherence

The question of what keeps people healthy has been asked continuously since its initial implementation by Antonovsky in 1979. The medical sociologist developed and pioneered the concept of salutogenesis (the development of health) which is anchored in its main concept, the SOC (Antonovsky, 1987).

The SOC is a basic life orientation which is connected to an individual’s sociocultural experiences across the lifespan. It consists of three sub-components, namely comprehensibility, manageability and meaningfulness (Lindström, 2006; Mittelmark and Bauer, 2017). Experiences of consistency, regularity and repetition lead to a high SOC, while experiences that are viewed as uncontrollable, uncertain and unpredictable might lead to a low SOC (Morrison and Clift, 2006). The sub-components are defined as the sense of comprehensibility (referring to how one understands the world), the sense of manageability (how one copes with challenges) and the sense of meaningfulness (how one is motivated and how one defines one’s meaning in life; Antonovsky, 1979, 1987; Mayer, 2011).

Individuals who have a strong SOC normally perceive their environment as predictable, consistent and stimulating (Mayer, 2011) and are found to be encouraging, engaging and good listeners (Mitonga-Monga and Mayer, 2020). They further believe that they are able to cope with challenging situations by activating their own resources. Additionally, they are able to see meaning in their lives and can manage their own emotions and motivations (Strümpfer, 1995) without feeling threatened or anxious when experiencing stressful events or situations (Braun-Lewensohn and Mayer, 2020).

According to Antonovsky (1987), women from lower societal backgrounds are at greatest risk of having a low SOC. Recent research has found that SOC is quite comparable across gender (Grevenstein and Bluemke, 2022). Gender and class differences, however, may play an important role in influencing SOC in its development (Lindström and Eriksson, 2005). For women especially, SOC seems to be reduced when women lack social support (Volanen, 2011). Although SOC is mainly developed in childhood, it continues to develop across the lifespan (Mayer, 2011) and it supports individuals in remaining healthy and in coping with complex or challenging situations.

## Faith development

The FDT was originally developed by James Fowler (Coyle, 2011) who was an American theologian and a Professor in Theology and Human development in the US. Fowler wanted to increase the comprehensibility of human values, meaning in life, and also belief in God (Fowler and Dell, 2004). Faith, according to Fowler (1986), is about creating meaning in life which might be religion-based, but does not necessarily have to include a religious or Christian belief system. It affects the orientation in life, changes over time (Fowler, 1984), and improves the understanding of extraordinary individuals (Runyan, 2006). According to the theory, individuals develop in three parts: the pre-stage during childhood, the lower stages (1–3) during childhood to adulthood, and the higher stages (4–6) in adulthood, although they might not be ultimately attained (Ashdown and Gibbons, 2012). The stages have been extensively described in the psychobiographies mentioned above and are therefore only summarised here (Fowler, 1981, 1984). Further, it can be highlighted that in these previous psychobiographies the assumptions of critical aspects of the theory have been discussed (e.g., Streib, 2003; Coyle, 2011; Ashdown and Gibbons, 2012; Mayer, 2017) which include, for example the critique of Coyle (2011) regarding Fowler’s definition of faith being broad and unspecific and aims to change from the idea of faith as being connected to God, towardsdefining faith as faith as a dimension of human meaning-making and understanding. Additional critical aspects are that Fowler’s theory is strongly influenced by his socio-cultural, gender and religious background (Baxter, 2006), while he also uses a sample that is mainly recruited in North America (Fowler, 1981). Slee (2004, 2021) has also recently pointed out that faith needs a gendered perspective – which is not addressed in Fowler’s theory. In summary, critical voices addressed shortcomings in the theory with regard to theoretical, structural methodological foundations (e.g., Coyle, 2011). The criticisms regarding Fowler’s theory were consciously taken into consideration in this study.

According to Fowler (1981, 1984) as for example cited in Mayer (2017), Stage 0 (Primal faith)^1^ occurs between ages of 1–3 years, and develops based on trust, loyalty and meaningful commitments with primary caregivers. Stage 1 (intuitive-projective stage) involves increasing meaning in emotional and perceptual ordering of experiences and faith, being based on symbols and images of visible power. Stage 2 (mythic-literal stage) provides a faith concept based on stories of self and other, where God is personalised and where goodness is rewarded and badness is punished. Here, the “11-year-old atheist” concept (Fowler, 1981) often brings disbelief in God and the idea of the individual in the context of the collective gains in importance. Increasing self-awareness and meaning are created in Stage 3 (synthetic-conventional stage) through relationship and role development, and the importance of faith and social perspectives. God is represented with personal qualities of acceptance and nurturing. Many individuals remain at Stage 3, while others move toward Stage 4 (individuative-reflexive stage, between 20 and 40 years) in which the individual authority is experienced within the self and personal values and belief are developed and reflected. At this stage, social relationships are evaluated, coherence of faith is built and boundaries are clarified. During Stage 5 (paradoxical-conjunctive stage), which hardly ever occurs before the age of 30, the individual develops multiple perspectives and acceptance of paradoxes. Faith is integrated with life as a deep belief. Own sociocultural boundaries are overcome and a new relationship with God is developed while meanings beyond one’s own traditions increase. A humble awareness is developed which then leads to the final stage, Stage 6 (universalising faith), in which individuals are viewed as whole individuals, independent of social class, gender, race, religion, nationality, age and political ideology. Here, tensions are embraced and transformed. An overall love for each and every person is experienced, and boundaries are overcome.

Generally, individuals can transition stages which are interconnected and which are viewed as flexible (Ashdown and Gibbons, 2012; Coyle, 2011; Mayer, 2017; Jones, 2022). Besides the stages, Fowler (1984, 1987) emphasises the importance of vocation in life which plays an important role in building the relationship with God. Vocation refers to the development of the personal identity and its vocation to find answers to who one is (in young adulthood), the aim to respond to questioning life’s vocation on deeper levels while waiting for God’s calling (middle adulthood), and finally to the setting of new priorities while dealing with one’s personal calling (older adulthood). The awareness of life’s vocation and spiritual transcendence refers to the relationship with God through caring for the environment (1), through God’s governance, justice, lawfulness and relationship-building (2), and finally, through God’s liberation from socio-economic and political ideologies and boundaries, bringing the fulfilment of feeling blessed by God.

## Research methodology

### Research design

The research methodology used in this article is qualitative in nature and defined as a psychobiography which is a biographical account which deals with the life of an extraordinary individual based on the use of a selected psychological theory (Ponterotto, 2017a; Mayer and Maree, 2018). According to Ponterotto (2017b), the life of a person can be uncovered and reconstructed by implementing the theory for analysing the life of the subject. The research paradigm is based on a hermeneutical-interpretative understanding (Creswell, 2013). The theories applied in this single-case research design (Yin, 2018) are salutogenesis and FDT.

### The sample

The sampling process in psychobiographies is anchored in the choice of a psychobiographical subject (Du Plessis, 2017). This study uses a non-probability, purposeful sampling approach, relying on the researcher’s judgement to determine the desired theory-based aspects of the life of the sample and ensuring that the analysis is rich and in-depth (De Vos et al., 2005).

ESH was selected for study by means of purposeful sampling because, as an extraordinary woman of the 18th and 19th centuries, she was well established, not only through her husband’s political career, but also through her own legacy. In her life she had to cope with various losses and tragic setbacks; however, she coped with them and conducted an impactful, long life in and for US society.

The researcher chose this subject based on her interest in women and her coping, sense of coherence and failth development in different socio-cultural, political and historical contexts. Thereby, her main focus of interest is the question how women cope in society and how they stay healthy and well while experiencing major life challenges. ESH seemed to be a very interesting person to be studied with regard to her life history and her ways to overcome challenges. The author is aware of the criticisms of both theoretical approaches which are used in this study and, e.g., that FDT was criticised as socio-culturally biassed and gendered. However, the theories used still seemed to be valuable to be used with regard to the subject since a lengthy literature analysis and interpretation showed that ESH’s life, faith development (FD) and SOC could be interpreted with regard to the stages and models, considering the critical aspects of it consciously.

### Data collection and analysis

Mainly secondary sources were used to explore and understand ESH’s life, her coping skills, SOC and faith in more depth (Du Plessis, 2017). The researcher used secondary sources, articles and biographies, especially of ESH’s husband, Alexander Hamilton (Allport, 1961; Fouché and Van Niekerk, 2005; Noack, 2021). As described, only publicly available data and information was used (e.g., Kenyes, 1931; Miller, 1959; Knott, 2002; Chernow, 2004; Miranda, 2015; New Netherland Institute, 2020). The study displays a low risk of ethical considerations (Noack, 2021).

The researcher’s interested in the subject started in 2016 with the release of the musical “Hamilton.” Over the years, she spend time reviewing literature on the subject chosen and the US-American context and then interpreting literature on ESH in the context of SOC and FDT. The researcher has worked on SOC since 2006 and on FDT since 2014 and could therefore read and interpret the literature on the life of ESH from a SOC and FDT perspective.

Mainly secondary literature – biographies, interviews, and articles (e.g., Presnell, 2004; History of American Women, 2022) – not only on ESH, but also on her husband (e.g., McDonald, 1979; Hendrickson, 1981; Emery, 1982) – was used due to the lack of primary literature, such as letters or autobiographies.

Data evidence was extracted, categorised, and analysed using SOC and faith as strategies to identify salience (Demorest, 2005; Yin, 2018). For the data analysis, thematic analysis was used to ensure hermeneutic interpretation and reconstruction of the life (Dilthey, 2002). The researcher familiarised herself with the data and theories. By doing so, different themes and categories emerged and the categories of the theories used were applied to and matched with the findings regarding the life of the subject. Surely, the interpretation is informed by the socio-historical context of the subject interpreted (Ponterotto, 2014; Fouché et al., 2018), however, the socio-historical context is not in the centre of this psychobiographical interpretation.

Methodological imitations with regard to the data analysis and interpretations include the lack of primary data and that often her life has been described through the lens of her husband’s life (e.g., McDonald, 1979; Hendrickson, 1981; Emery, 1982).

### Quality criteria and ethical considerations

Ethical guidelines for psychobiographies were followed throughout the study (Ponterotto and Reynolds, 2019). To ensure ethical standards were maintained, only data from freely accesible public domains was used (Ponterotto et al., 2017). Founded on ethical considerations, the study is intended to contribute to learning about the life of extraordinary individuals and their deeper understandings (Ponterotto and Reynolds, 2019). ESH is explored in this study in the most ethical, empathetic, accountable and respectful way (Schultz, 2005; Ponterotto, 2015). This ethically founded approach aims to contribute to expanding the public knowledge of ESH and her ability to cope with life’s challenges, based on her SOC and her faith. As described by Wegner (2020), potential harm to the researched individual is therefore avoided.

The study is built on trustworthiness to enhance consistency and reliability (Creswell, 2013). Triangulation of data was applied to enhance trustworthiness and rigour, as previously done in psychobiographies (see Du Plessis, 2017; Mayer, 2021b). The researcher engaged with the biographical data and the life history of the subject and created a conscious awareness of the subject, as well as of the relationship of the subject and the researcher (Ponterotto and Moncayo, 2018). The subject’s life and contexts were investigated and facilitated through the analysis and interpretation of the subject’s unique life and living contexts (Ponterotto, 2014). Finally, the researcher considered the connection of the methodological competence and ethical considerations in psychobiography (Ponterotto and Reynolds, 2019). The eight best practices of Ponterotto (2014) were taken into consideration, e.g., using thick descriptions, including an understanding of the socio-cultural and historic context, conducting an accurate and balanced assessment, and keeping the researcher’s bias and horizon and understanding into consideration, as well as considering various ways of interpretation and considering alternative explanations during interpretations.

Although this study provides a deep insight into selected particular aspects of ESH’s life, it also has limitations. The research is descriptive and idiographic, using the unit of analysis as being a single case study (Fouché and van Niekerk, 2010) which is analysed holistically (Carlson, 1988), and the researcher used English and German literature from the public domain for analysis. Finally, the interpretations of the researcher might have led to a subjective bias (Yin, 2018) which could be informed by the researcher’s nationality, her cultural background, her gender and her professional background. The researcher is female, of German origin, living in South Africa and strongly interested in women leaders and the roles of women in leadership in different cultural contexts. The potential bias has been addressed through self-reflection and inter-subjective validations (Yin, 2018) in discussions with other psychobiographical and qualitative researchers.

## Findings and discussion

In the following section, the psychobiographical findings are presented with regard to the integration of the SOC and development of faith in ESH’s lifetime (9 August 1757 to 9 November 1854). She was born in Albany in the US state of New York, the daughter of Philip Schuyler and Catherine Van Rensselaer Schuyler (Presnell, 2004; Roberts, 2010). Both of her parents were among the most wealthy and politically influential families in the American colonies who had arrived “from the Netherlands and had been well established in New York since the mid-1600s” (Reiser, 2017, p. 14). Her mother came from an established, wealthy and influential family background. Her father was a revolutionary war general and shared similar political interests with Alexander Hamilton, whom ESH first met at her Aunt’s house when she was 22 years old in 1780. They were married within the same year and according to one of ESH’s letters, their love grew throughout their marriage (American Experience, 2022).

During her marriage to Hamilton (between 1780 and 1805), ESH was described as being sickly, suffering from nervous attacks and many miscarriages. She gave birth to numerous children of whom eight survived (Flexner, 1977). While she felt strained during their marriage owing to her husband’s infidelities, the loss of their eldest son and various socio-economic crises, she improved in health after Hamilton’s death and lived until she was 97 years old (Flexner, 1977).

In the following the life, SOC and faith of ESH will be presented in chronological order from childhood to old age. An overview of the development of her SOC will be provided in Table 1; a summary of her FD will be shown in Table 2, and her vocation in Table 3.

**TABLE 1:** Sense of coherence (SOC) in ESH’s life perspective.

SOC	In life development perspective
Comprehensibility	Predominant in childhood and teenage years: –Understanding the world in its socio-economic and political complexity
Manageability	Predominant during her marriage: –Managing marriage, life, children –Managing husband’s career and political moves
Meaningfulness	Throughout her life, but predominant after her marriage: –Through faith –By establishing her husband’s legacy –By restoring her husband’s reputation and life –Through children and social contacts –Through building an orphanage and a school across social class barriers

**TABLE 2:** Faith development (FD) in ESH’s life.

Lifespan	FD stages of ESH						
	Stage 0	Stage 1	Stage 2	Stage 3	Stage 4	Stage 5	Stage 6
Childhood	x	x	x				
Teenage years			x	x			
Her twenties				x			
Her thirties				x	x		
Her forties				x	x	x	
Her fifties				x	x	x	
Her sixties					x	x	
Her seventies					x	x	
Her eighties					x	x	
Her nineties					x	x	

**TABLE 3:** Vocation in Elizabeth Schuyler Hamilton’s (ESH’s) life.

Vocation in life	Throughout ESH’s lifespan
God’s creation and caring for others and the environment	• Caring for her children and husband • Helping others in more challenging positions • Building an orphanage • Building a school
God’s governance and his justice and lawfulness within societies	• Supporting her husband’s politics
God’s liberation from socio-economic and political ideologies and boundaries	• Keeping her political ideologies and boundaries, but overcoming her socio-economic boundaries by marrying Hamilton (lower socio-economic class) • Supporting children from lower socio-economic classes

### Childhood and teenage years

ESH grew up in a very elegant mansion in Albany and was raised by one of the wealthiest families in the US (Chernow, 2004). During childhood and teenage years, ESH did not receive much formal schooling and had only received some tutoring (Chernow, 2004). However, as the daughter of a soldier, general and statesman, she was educated in political and public affairs (Chernow, 2004). When she was 13, her father took her to a conclave of “Chiefs of the Six Nations” at Saratoga, where she was given an Indian name, “One-of-us” (Ogden and Meredith, 1980, p. 9). Through the political affairs of her father, she must have developed a broader understanding of politics, the military, societal relations and military actions (*comprehensibility*). ESH was described as being deeply devoted to her Christian faith (People and Events, 2007; *meaningfulness*), warm, strong-willed, robust, empathetic and delightful (Chernow, 2004).

### The marriage

ESH met Alexander briefly in her teenage years and met him again when she was 22, shortly afterward marrying him. According to Smucker (1858):

The bride was beautiful, accomplished, talented, and well-born. Her vivacity, intelligence, and amiability, had rendered her a universal favourite in the polished circles of Albany, at that time one of the most select and cultivated towns in the country.

The marriage to Alexander Hamilton took place in 1780. McLane (1911, pp. 95–96) points out that her main “talents were domestic and she is best remembered as a loving wife and devoted mother.” Kaveney (2018), however, counter-argues that ESH represented immense strength by demonstrating character development throughout her life, going beyond the expectations of women during that period.

ESH supported her husband and counselled him with regard to his political affairs, and helped him to manage his papers (Wilson, 2018). Chernow (2004, p. 130) states that ESH did “everything in her power to focus the spotlight exclusively on her husband” (*manageablity*).

During her marriage, ESH conducted close friendships with her husband’s friends’ wives, such as Martha Washington, the wife of George Washington (Griswold, 1867). So she seemed to have a network of friends and kept close family bonds with her parents and siblings (Chernow, 2004).

ESH gave birth to eight children and had several miscarriages. Accordingly, she displayed a strong meaningfulness with regard to her family roles and her personal interests in supporting her husband and bringing up their children (*meaningfulness*).

When Hamilton joined the army, they had already six children. ESH was unhappy with his new career path, since he had lost income from his private law practice and was not paid for several months after he joined the army (Harper, 2004). However, she managed, and he promised to build a house for them, called “The Grange” in the countryside (*manageablity*). When Alexander Hamilton had an affair and after the revelations in 1792 and again in 1798 that Alexander was involved in a love affair, ESH suffered (Harper, 2004) and seems to have been deeply shocked. However, she was “determined to cope, in her typically Spartan way” (Harper, 2004, p. 204; *manageability*).

In the year 1801, life became tragic for ESH when her younger sister Peggy died aged 42 and her eldest son Philip was shot in a duel (Reiser, 2017). She then gave birth in 1802 to her last child, called “Little Phil,” in honour of his late brother. In 1803 ESH’s mother died suddenly and in 1804 her husband was wounded in a duel against Aaron Burr, who was the vice president at that time (Chernow, 2004). Shortly, after the death of her husband, her father died and she had to cope with all of these losses within a short time, finding peace in life through God (Reiser, 2017; *meaningfulness*).

### Life after her husband’s death

After her husband’s early death, caused by a duel with his political rival, ESH managed to a degree to rescue her husband’s historical reputation and aimed to restore it for the rest of her life, being “committed to one holy quest above all other: to rescue her husband’s historical reputation” (Chernow, 2004, p. 2). To cope with his early death, ESH founded two institutions in New York to support lower-income children, the Hamilton Free School and the Orphan Asylum Society, the first orphanage in New York (Kiger, 2020). According to Mazzeo (2019), ESH was passionate about the welfare of children and she focused on finding solutions throughout her life as soon as problems occurred (*manageability*). Reformational and charity schools have been discussed critically in the United States of America and represent the moral ideas of “upper-class New Englanders in response to population growth, immigration, and other social changes” and the moral and righteous society (Encyclopedia, 2022), ESH’s perspective was most probably partly influenced by these ideas and value sets, which can be viewed as critical. However, her engagement might have also been anchored in her own experience of growing up without a formal schooling (Acton Institute, 2016) and her idea to give this opportunity to others and contribute to a better life.

ESH had a strong faith, purpose and meaning throughout her life. She was described as having “a sharp intelligence, a fiercely indomitable spirit, and a memory that refused to surrender the past” (Chernow, 2004, p. 1). Chernow (2004) describes her life as passionate and deeply religious and based on the humanist idea that all children should be literate (*meaningfulness*). Her strong faith in God helped her to overcome challenges and obstacles in her own life and to deal with crises while helping others in more challenging positions, such as orphans and individuals from lower classes.

After the death of her husband, ESH’s purpose in life was multifold (*meaningfulness*): she aimed to elevate her husband’s reputation, created his biography – which was later published by her son, John C. Hamilton – and engaged in social causes such as building the first orphanage for children in New York (Flexner, 1977). In addition, ESH focused on her connection to her children, which kept her alive and contributed to her meaningfulness (Kiger, 2020). She further derived a powerful meaning in life by helping children and families from lower-income classes to receive education. She managed to do so by activating women from wealthier families who supported her projects financially (Kiger, 2020). Reiser (2017, p. 99) points out that the story of ESH has been told in different ways, but that only recently has it been acknowledged that she played an important part in the American consciousness, changing from a “stereotypical widow of the late 18th and early 19th centuries to a robust and powerful keeper of her late husband’s legacy.”

Table 1 presents a summary of the development of her SOC in terms of the sub-components of sense of comprehensibility, sense of manageability and sense of meaningfulness which developed across her lifespan.

### Faith development across her lifetime

ESH grew up in a religious family with a Christian background. Her upbringing therefore reflects Fowler (1981; 1984; 1986) approach that is anchored in the Christian belief. It may be assumed that ESH grew up with her primary caregiver at home, particularly since she did not attend formal schooling, but was instead educated by her parents. Her parents therefore most probably represented her primary approach to her faith and fostered her strong belief (Stages 0–3) which was based on trust, loyalty, meaningfulness and commitment (Stages 0 and 1). She was known as “the little saint” (Acton Institute, 2016) and was described as stoic, religious and averse to self-pity (Chernow, 2004). She was considered to be “good-natured though somewhat serious […] at ease in the outdoors and devout in her Christian faith” (American Experience, 2022). She was a member of the Dutch Reformed Church and it was important to her to bring up her own children with strict religious instruction (Acton Institute, 2016). Part of this religious approach to life was reading from the Bible every morning, which reflects Stage 3 of the FDT. ESH’s developed passionately her role as a wife and mother (Stage 3).

For ESH, Stage 4 of the FDT is represented quite clearly in her life since her personal values reflected her focus on social relationships and her strong desire to nurture and care for others. She demonstrated deeply held values in fostering relationships and clarifying her own boundaries. For example, she struggled for many years with her husband’s infidelity and his love affair, but she managed to overcome it and stay with him by redefining her personal boundaries, the strengthening of her values and her self-coherence (Stage 4).

After the death of her husband, ESH redefined her sociocultural boundaries by opening up an orphanage and by developing a meaning beyond her own traditions (Stage 5).

ESH did not reach Stage 6 which proclaims the universalising faith, in which individuals are viewed as independent of social class, gender, race, religion, nationality, age and political ideology. On the contrary, ESH retained the focus on her political, national and religious ideologies and it was not her personal aim to broaden them. However, it might be highlighted that she carried an overall love for humanity and a general non-violence through which her boundaries were overcome.

Table 2 provides an overview on the FD across ESH’s lifetime.

### Vocation in life

It is appropriate to assume that ESH’s identity and strong meaningfulness were anchored in her upbringing, which was based on education and developing a deeper understanding of the world, and the complex sociopolitical circumstances as seen through her parents’ eyes (Mazzeo, 2019). ESH, growing up in a wealthy and influential American family, aimed to develop herself, trying to find answers to who she was. Various literature accounts highlight that she was very settled in her own identity, aware of her abilities, interested in social activism with a philanthropist mission (Presnell, 2004; History of American Women, 2022), and showed strong spirit (Esme, 2022). It seems that, from a very young age, ESH was self-aware and self-conscious and her belief and faith was anchored in God and shaped by political insights (Chernow, 2004; People and Events, 2007).

During her marriage and middle adulthood, ESH supported her husband and brought up her children (Chernow, 2004). From the death of her husband onward, ESH set new priorities, managed to pay off the debts of her husband, bring up her children and support social activities, such as building the first orphanage and the first school for children from lower social strata.

It appears that ESH’s life was fully guided by her belief in God. According to Fowler (1981, 1984), the vocation in life and spiritual transcendence refer to three different aspects in the relationship with God: through caring for the environment, through God’s governance, justice and relationship-building, and finally through God’s liberation from socio-economic and political ideologies and boundaries, leading to fulfilment in feeling blessed by God. An analysis of the life of ESH reveals that care for the environment and others in the environment was of major importance across her lifespan, particularly in caring for her children, caring for her husband and his career, building the orphanage and the school and helping others in more challenging situations (Kenyes, 1931; New Netherland Institute, 2020).

With regard to God’s governance, justice, lawfulness and relationship-building, ESH’s vocation is mainly represented in supporting her husband’s political career, standing in for certain political views and perspectives and reflecting them back to her husband, supporting him in writing his political attempts (Chernow, 2004).

Finally, she also found part of her vocation in life in God’s liberation from socio-economic and political ideologies and boundaries to a certain degree. In one respect, she overcame socio-economic boundaries by marrying Hamilton who was an orphan coming from a lower-class socio-economic background. On the other hand, she aimed to overcome these boundaries by helping children from lower-class socio-economic backgrounds with education and by giving them a home in the orphanage.

It may be summarised that her relationship to God was essentially influenced by God’s creation and caring for others within her family and beyond. This was particularly true until her middle adulthood. Later, ESH mainly focused on diminishing the boundaries of people from different classes by providing a home and education for them. However, the focus of her actions was always based on the foundation of human values and care for others.

## Conclusions and recommendations

This study aims to contribute to psychobiographical literature on exploring the coping mechanisms of women from diverse backgrounds. Coping, in this study, was understood as being supported by SOC and FD throughout the lifetime. The subject of the study was ESH, the wife of Alexander Hamilton, one of the founding fathers of the US, who lived in the 18th and 19th centuries.

The study shows that ESH had a strong SOC which helped her to cope with a number of difficult situations and losses throughout her lifetime. Coming from a wealthy, educated and well-established Christian family background, she developed keen comprehensibility and meaningfulness during her childhood and teenage years. During her marriage to Hamilton, she showed great strengths in managing her marriage and children, her husband’s politics, his extramarital affairs, and the losses she had to cope with. She drew on all of the resources she had (family, friends, and a firm faith in God) to deal with and overcome her challenges. After the loss of her husband she managed to cope with the financial strain and all of her children on her own, mainly through her own sense of meaningfulness and a strong belief in God. It may be assumed that her sense of purpose and meaningfulness was in particular influenced by her belief in God and her desire to contribute positively through the life of others, such as her late husband’s career and reputation and her support of children from lower socio-economic classes.

ESH’s faith developed throughout her life in terms of Stages 0–5, as according to Fowler’s FDT. Although ESH had a love for humanity and non-violence, she did not overcome any religious or national boundaries. She stayed firmly within her Christian belief system, and because the US was in the process of being created, she did not aim to overcome any brand-new national boundaries.

Finally, one may conclude that her relationship with God, as described by Fowler (1981, 1984), was mainly based on caring for others and doing good, although she also supported her husband’s politics and lawfulness as well as liberation from sociocultural ideologies.

For future research and practice, it is recommended that future psychobiographies should take the exploration of the lives of women into consideration, thereby exploring women’s lives during different times and within various cultural contexts. It is particularly important to explore women’s coping mechanisms throughout their lifetime. These coping mechanisms can be explored through theories such as salutogenesis and SOC, as well as FDT.

It is also important to highlight that FDT needs to be explored specifically in terms of context. This study shows that, for example, Stage 6 of the FDT might not be reached owing to the contextual situation of the individual whose life is explored. Therefore, future psychobiographical research should aim to further develop theories, such as in this case, SOC and FDT.

On a practical note, psychobiographies on the coping mechanisms of women can be referred to in counselling and therapy as positive examples of extraordinary women coping with difficulties and challenges throughout their lifetimes, in different sociocultural and contextual situations. This psychobiography of ESH can also be used to provide an example of how an individual can deal with tragic losses and return with resilience to contribute positively to the lives of different individuals and to society in general.

## Data availability statement

The original contributions presented in this study are included in the article/supplementary material, further inquiries can be directed to the corresponding author.

## Author contributions

The author confirms being the sole contributor of this work and has approved it for publication.

## Footnotes

1 It needs to be taken into account that different theoretical approaches to FDT deal with stages, life phases and age ascriptions in different and varying ways. Discrepancies can be recognised in the literature (e.g., compare Fowler (1984; Fowler and Dell, 2004; Coyle, 2011), as acknowledged in Fowler et al. (2004).
